# Psychological Strategies and Protocols for Promoting School Well-Being: A Systematic Review

**DOI:** 10.3389/fpsyg.2022.914063

**Published:** 2022-06-09

**Authors:** Pierpaolo Limone, Giusi Antonia Toto

**Affiliations:** Learning Science Hub, Department of Humanities, University of Foggia, Foggia, Italy

**Keywords:** wellbeing, school, dropout, deviance, addiction

## Abstract

Physical, socio-economic, cultural and mental challenges faced by students have been associated with adverse impacts on school wellbeing, resulting in increased school dropout and deviant behaviour. This systematic review has analysed the present knowledge on factors associated with school dropouts to identify psychological interventions for promoting school wellbeing. A systematic search was done of the ScienceDirect, APA PsycINFO, Emerald and Google Scholar electronic databases. A hand-search was also done of the reference list of the included studies. The initial search resulted in 448 studies, and the search of the references list of the considered studies resulted in 28 more articles. The application of the eligibility criteria resulted in the inclusion of 38 studies in the review. The study established several factors associated with school dropouts and social deviance, such as school climate, school structure, and those defining social interaction among students. Mental and emotional health was identified as the main factor influencing school dropout and social deviance. A positive school climate should be the primary consideration for promoting school wellbeing. School administrations, teachers, and parents should collaborate to positively improve conditions in schools.

## Introduction

School completion rates have shown a marked improvement over much of the past century, rising from single digits at the turn of the 20th century (Ramsdal and Wynn, 2022). This shift has been associated with educational changes such as the standards movement in education, social activities and cultural changes. Nevertheless, the dropout problem has endured through these changes, even amid higher school completion rates (Dupéré et al., 2015). School dropout has considerable consequences, including perverse implications on employment, lifetime earnings, and health literacy. Students often fail to complete high school for complex reasons that manifest earlier in their lifetimes (Dupéré et al., 2015; Krane et al., 2016).

School dropout rates are linked to physical and mental problems, substance abuse, antisocial behaviour, negative school attitudes, low quality of education, parenting problems and family challenges (Ramsdal and Wynn, 2021). These factors can be shown to have an initial impact on school wellbeing, which often leads to school dropout. Nevertheless, the uniqueness of these factors necessitates a multifactorial approach for risk and needs assessment to promote the creation of interventions aimed at mitigating school dropout (Gubbels et al., 2019).

School dropouts self-report various reasons for leaving school, yet these do not accurately construct the picture of the underlying problem. It is usually difficult to establish a causal relationship between any single factor and the decision to quit school 5. However, preliminary studies have provided a framework that delineates the factors associated with students’ individual characteristics and elements related to the institutional aspects of their families, schools and communities (Rumberger and Lim, 2008). The latter category encompasses school wellbeing, which is correlated to school dropout rates. According to Doll et al. (2013), dropping out is the culmination of a much longer process of leaving school, beginning long before the day that a student eventually ceases attendance. However, Doll et al. (2013) identified a different framework for the analysis of factors influencing dropping out of schools. The framework constitutes push, pull and falling out elements, and while each type of dropout antecedent has credence, pull factors demonstrated the highest rates (Bryk and Thum, 1989; Doll et al., 2013).

The key difference between push, pull and falling out factors is agency. In push factors, the school is the agent whereby a student is removed from school due to a consequence. In pull factors, the student is the agent, such that attractions or distractions lure them out of school. In terms of falling out elements, circumstances exist that neither the school nor the student can remediate, and as a result, the connection students have with school gradually diminish (Doll et al., 2013). Dropping out factors are associated with the characteristics of the individual students—their educational performance, behaviours, attitudes and backgrounds—as well as the characteristics of the families, schools and communities where they live and go to school. However, Rumberger and Lim (2008) determined that no single factor can entirely explain a student’s decision to continue school until graduation.

According to Rumberger and Lim (2008), dropouts have higher rates of unemployment, lower earnings, poorer health and higher rates of mortality, higher rates of criminal behaviour and incarceration, as well as increased dependence on public assistance compared to graduates. Still, Drapela (2004) established that dropping out of high school has no substantive effect on later drug use. This relationship was assessed with two fundamental measures of association, zero-order correlations and partial correlations. Deviant behaviours such as post-dropout drug use, as measured by tobacco, alcohol, and marijuana consumption, were shown to have statistically significant but non-substantive relationships to dropping out among dropouts (Croninger and Lee, 2001; Drapela, 2004). According to Fernández-Suárez et al. (2016), alcohol abuse and substance use have direct consequences on individual characteristics related to deviant behaviours. Furthermore, significant dropout risk associated with poor mental health majorly occurs in vocational and higher education (Rumberger and Rotermund, 2012; Hjorth et al., 2016).

To address the dropout crisis requires a better understanding of why students drop out; however, identifying the causes of dropping out is extremely difficult (Rumberger and Lim, 2008). The dropout problem is considered a multifactorial phenomenon resulting in an emphasis on and development of school-wide multi-component interventions and strategies, mainly based on school wellbeing research (Johansson and Uhnoo, 2019). School wellbeing constitutes factors relating to school characteristics, the school as an organisation, the school climate or culture, and the collaboration of professionals in the school. These factors include inadequate or inefficient disciplinary frameworks, poor academic climate and low school attachment, and conflicts. According to Gallup (2017), a significant solution to school dropping out would be for governments to allocate more funds to districts that report alarming rates. This intervention would attract higher-quality teachers to the area who, in turn, are better suited to motivate students to stay in school and complete their education.

According to Lee-St. John et al. (2018), drop out intervention strategies research should go beyond the typical school boundaries to mitigate dropout risk factors. Still, schools cannot achieve outreach independently and will require significant, meaningful and effective partnerships with community agencies. However, the barrier to research regarding dropout intervention strategies is that many interventions that can be comprehensively evaluated are narrow in focus and modest in scope. Moreover, complex interventions that address the comprehensive needs of students at risk of dropout can be challenging to study (Lee-St. John et al., 2018). The purpose of this systematic review is to examine the current state of knowledge regarding the risk factors associated with school dropping out and deviant behaviours and identify interventions used to prevent dropouts, as well as their outcomes and effectiveness.

## Methods

### Literature Search and Reporting

This research paper has been reported based on the Preferred Reporting Items for Systematic Reviews and Meta-Analyses (PRISMA) 2009 guidelines. A systematic literature searches until 27 March 2022 was carried out over multiple electronic databases, including PubMed, Google Scholar, ScienceDirect and CINAHL. A hand search of the reference lists of the studies obtained in the initial search was also conducted to maximise the scope of the data search. To ensure that the most cited and recently published articles were obtained, results on the first two pages of the databases were majorly considered. A list of terms was formulated for the research questions, and Boolean operators were used to group phrases and keywords. The following keywords were used in the search process: (“teacher-student relationship” OR “school wellbeing” OR “school climate” OR “student wellbeing”) AND (“dropout OR misbehaviour OR deviance OR defiance); all the sources were published between 2000 and 2022.

### Guidelines and Selection Criteria

To select relevant studies, several inclusion and exclusion criteria were formulated. The eligibility criteria were such that they allowed the comprehensive examination of psychological protocols and strategies that minimise social distress and promote school wellbeing, while ensuring the reporting of quality findings. The studies also had to focus on the students’ lives in school. Additionally, articles were also included if they reported on correlated factors associated to a student or a teacher. Consequently, studies were excluded if they did not include as participants were students or teachers, and if they did not empirically evaluate the relationships between factors in the school environment, dropout and social deviant behaviours.

The titles of the articles obtained in the primary search were analysed to ensure they discussed the subject under consideration. Following this process, the included articles were subjected to abstract screening, which resulted in the elimination of more articles. The articles included after the title and abstract screening needed to have answered the research questions. Subsequently, the remaining articles were then subjected to a full-text reading to examine their level of evidence and determine their significance in this systematic review.

### Assessment of Methodological Quality

The articles were subjected to methodological quality assessment prior to the data extraction process; all the articles had to pass the criteria to be considered for data extraction. The assessment items were clarity in stating the research question, participant sampling method information, study design, data collection methods, data analysis, study limitations and comparison to the existing literature on the research topic. The studies also had to compare study findings with existing literature. Articles that comprehensively discussed these aspects and followed the criteria were considered high quality.

### Data Extraction and Synthesis

Data from studies that passed the eligibility criteria were extracted into pre-defined descriptor tables. The tables collected information related to the following aspects: author, year, study design, number of participants, objective statement and study findings. Research findings were examined using a reciprocal translation approach and primarily involved considerations of psychological protocols and/or strategies for promoting school wellbeing.

## Results

### Search Results

The initial database search yielded 448 citations and reference list search resulted in eight citations. The 244 articles that remained after elimination of duplicates were subjected to title and abstract screening. Finally, the application of the eligibility criteria resulted in the inclusion of 38 articles.

## Summary of Results

Results were organised according to the main themes emerged from the analysis of the included studies. Specifically, below are reported the risk and protective factors that were found leading to school dropout and social deviance in each article. In addition to this, summary of results related to the interventions found were reported below (Tables 1, 2).

**Table 1:** Study descriptor table.

Author and year	Study design	Region	Number of students
Archambault et al., 2009	Longitudinal study	Quebec, Canada	11,827 high school students
Bergeron et al., 2011	Case study	Quebec, Canada	2,360 secondary school students
Christle et al., 2007	Case study	Kentucky, United States	196 high schools
Frostad et al., 2014	Retrospective study	Norway	2,015 upper secondary students
Gottfredson et al., 2005	Retrospective study	United States	254 public secondary schools
Lee and Burkam, 2003	Retrospective study	United States	3,840 students
Lessard et al., 2010	Exploratory case study	Quebec, Canada	4,312 high school students (2,227 girls and 2,085 boys)
Wang et al., 2013	Longitudinal study	United States	1,400 students
Welsh, 2001	Retrospective study	United States	4,640 middle school students
Barile et al., 2011	Longitudinal study	United States	7,779 students
Hess and Copeland, 2001	Case study	United States	92 students
Kelly et al., 2021	Case study	Florida, United States	109 students
LaRusso et al., 2007	Retrospective study	United States	476 adolescent students
Loukas et al., 2006	Retrospective study	United States	489 students
Loukas et al., 2010	Longitudinal study	Central Texas, United States	476 adolescent students
Murray and Malmgren, 2005	Randomised control study	United States	48 African–American students
Ryan and Patrick, 2001	Longitudinal study	United States	233 students
Temple et al., 2000	Prospective study	Chicago, United States	1,159 African–American and Hispanic students
Piñeiro-Cossio et al., 2021	Review	European countries, United States and United Kingdom	10,357 students aged 7–18
Johns et al., 2019	Symposium	Chicago	40 experts bringing in the needs of schools and families
Marx et al., 2017	Review	Canada, United States, Northern Israel, New Zealand, Croatia and Southern Brazil	297,994 secondary school students
O’Reilly et al., 2018	Review	United Kingdom, Australia, USA, Sweden, Denmark, Germany, Ireland	9,700 students aged 5–19
Hou et al., 2021	Cross-sectional study	Australia	1,392 students aged 12.7–16.24
Gobat et al., 2021	Case study	Wales	22 secondary school students
Littlecott et al., 2019	Case study	Wales and United Kingdom	About 3,800 school students
Fernandez and Benner, 2022	Longitudinal study	United states	1,010 primary to second grade school students
Chan et al., 2022	Case study	California	55,383 first and second grade students
Saleem et al., 2022	Case study		
	United States	440 students university students	
O’Donnell et al., 2022	Longitudinal study	United States	294 secondary school students
Austin et al., 2022	Interview	United States	75 students with an average age of 11.6 years
Coetzee et al., 2022	Interview	South Africa	22 students of the age group 10–15
Fu et al., 2022	Case study	China	496 teachers from special education schools
Salceda et al., 2022	Focus group	Spain	13 Teenagers age group 15–18
Tsukawaki and Imura, 2022	Ethnography	Japan	500 primary and first grade students
Cittone and Villani, 2019	Book chapter—review	Europe	Children in age preschool
Song, 2021	Review	Various Afferents	Not specified
Zheng, 2021	Review	Varies Afferents	Not specified
Hunter et al., 2022	Experimental study	Colorado	18 junior high school teenagers

**Table 2:** Study descriptor table.

Author and year	Objective statement	Wellbeing factor	Theme
Archambault et al., 2009	To assess the contribution of student engagement to school dropout	Students engagement and its specific dimensions	School dropout
Bergeron et al., 2011	To examine the association between STR and achievement motivation with a student’s intention to dropout	Teacher–student relationship (STR)	Student’s intention to drop out of school
Christle et al., 2007	To examine the relationship between school characteristics and dropout rates	School size, student body, student’s ethnicity, academic achievement, attendance rate, suspension rate, school-law violation rate	School dropout
Frostad et al., 2014	To assess the relationship between social participation and motivation to leave school	Social participation	School dropout
Gottfredson et al., 2005	To explore the association between school climate characteristics and school crime and disorder	School’s organisational characteristics	School crime and disorder
Lee and Burkam, 2003	To explore the relationship between a school’s structure and organisation and a student’s decision to dropout	Learning curriculum, sector and size, and STR.	Student’s intention to drop out of school
Lessard et al., 2010	To analyse the relationship between students’ school wellbeing and intention to dropout	Commitment, achievement, satisfaction	Student’s intention to drop out of school
Wang et al., 2013	To investigate the relationship between STR and adolescent depression and misconduct	STR	Behavioural problems in students
Welsh, 2001	To explore the effects of school climate and student characteristics on school disorder	School climate and student characteristics	Behavioural problems in students
Barile et al., 2011	To investigate associations between teacher evaluation and reward policies, and student performance and dropout	STR climate	Effects of teacher evaluation and reward policies
Hess and Copeland, 2001	To investigate the relationship between coping strategies for stress and rates of finishing school.	Stress-coping strategies	Dropout rate
Kelly et al., 2021	To investigate the efficacy of psycho-spiritual education on school wellbeing and school climate	Mentoring program teaching psycho-spiritual principles	School wellbeing and perceived school climate
LaRusso et al., 2007	To examine the implications of a respectful school climate on student drug use and depression	School climate (support from teachers)	Student drug use and depressive traits
Loukas et al., 2006	To examine if the school climate is associated to adolescent conduct problems through school connectedness	School climate (interaction and competition among students and satisfaction with classes)	Student conduct problems
Loukas et al., 2010	To examine the contributions of early school connectedness to adolescent behaviour problems	School connectedness (social relations)	Student conduct problems
Murray and Malmgren, 2005	To examine the effects of increasing adolescent–teacher relationship	STR	Effects of improved STR
Ryan and Patrick, 2001	To investigate the relation between school social environment and students’ motivation and engagement in school	Class social environment	Student motivation and engagement
Temple et al., 2000	To investigate the effects of participation in the Chicago Child–Parent Centre and Expansion Program on school dropout	Early childhood intervention	Problematic behaviour and dropout rates
Piñeiro-Cossio et al., 2021	To analyse interventions for the improvement of psychological wellbeing at school	Activities physics	Wellbeing school
Johns et al., 2019	To discuss the findings of the Symposium on Protective Factors for LGBTQ Students	School climate, supporting educators, student identity	Protective factors for LGBTQ students
Marx et al., 2017	To evaluate the effects of postponing the start of lessons to support health, education and wellbeing in secondary school students	Postponing the beginning of lessons	Start time of lessons and hours of sleep for increased wellbeing
O’Reilly et al., 2018	To identify those interventions that can support the promotion of students’ mental health	Internal cooperation of the school community	School interventions for wellbeing at school
Hou et al., 2021	To improve wellbeing literacy to increase wellbeing	Literacy on welfare	Literacy and wellbeing at school
Gobat et al., 2021	For formative and pragmatic evaluations of the educational process to promote school wellbeing	Mapping of the socio-cultural and political contest	Promoting wellbeing through a restorative practice approach
Littlecott et al., 2019	For understanding the social interactions of school staff to foster student wellbeing	Interactions mediated by social networks	Role of school staff and social network on student welfare
Fernandez and Benner, 2022	To propose coping strategies to reduce the malaise resulting from discriminatory treatment in educational disparities	Coping strategies	Discriminations in educational disparities
Chan et al., 2022	To assess the support students receive and the perceived degree of wellbeing	Family, peer and school support	Support and wellbeing in school
Saleem et al., 2022	To assess the protective effect of racial-ethnic socialisation on ethnicity-related stress	Ethnic-racial socialisation	Ethnic-racial socialisation to reduce the negative effects of stress related to ethnic differences
O’Donnell et al., 2022	To test a longitudinal model of promoting confidence in adults and psychological wellbeing among adolescents	Positive expectations from adults	Promotion of student welfare through adult support
Austin et al., 2022	To evaluate the relationship between racial-ethnic connectedness and behavioural and emotional problems	Racial-ethnic connectedness	Effects of racial-ethnic connectedness on the wellbeing of African-American students
Coetzee et al., 2022	To evaluate the usefulness of mental health programs for reducing symptoms of anxiety and depression	Mental health programmes	Positive effects of mental health programs on symptoms of anxiety and depression in students
Fu et al., 2022	To study the relationship between social support, self-efficacy and the perceived wellbeing of teachers	Social support	Positive effects of social support on well being
Salceda et al., 2022	To analyse the effects of a Dialogic Literary Gatherings intervention on well-being and school performance	Meetings literary dialogical	Dialogic Literary Gatherings intervention to promote wellbeing and academic achievement
Tsukawaki and Imura, 2022	To assess the type of humour that has positive effects on students’ mental health	Teachers’ humour	The effect of teachers’ humour on student wellbeing
Cittone and Villani, 2019	To allow the revision work carried out to identify the positive effect of psychomotor intervention on multiple areas of development	Psychomotor intervention	The positive effect of psychomotor intervention on movement, cognition and emotions
Song, 2021	To investigate the effects of teachers’ optimism and effectiveness on student wellbeing	Optimism and effectiveness of teachers	The effect of teachers’ optimism and effectiveness on student wellbeing
Zheng, 2021	To assess the importance of teacher support on student wellbeing	Quality of the STR	The importance of teacher support on student wellbeing
Hunter et al., 2022	To assess the effects of culturally rooted afterschool programmes on students’ self-esteem, resilience and cultural identity	Cultural rootedness of planned afterschool programmes	The effect of culturally rooted afterschool programmes on students’ self-esteem, resilience and cultural identity

### School Dropout

Studies that evaluated the risks of dropping out were Temple et al. (2000), Hess and Copeland (2001), Lee and Burkam (2003), Christle et al. (2007), Archambault et al. (2009), Lessard et al. (2010), Bergeron et al. (2011), Frostad et al. (2014), Austin et al. (2022) and Saleem et al. (2022). Different studies evaluated the effects of different school climate factors and student characteristics on dropout risks.

### Student–Teacher Relationships

Most studies found a negative association between student–teacher relationships (STR) and the risk factor of dropping out (Lee and Burkam, 2003; Murray and Malmgren, 2005; Barile et al., 2011; Bergeron et al., 2011; Wang et al., 2013; Frostad et al., 2014; Littlecott et al., 2019; Song, 2021; Zheng, 2021). This means that in schools where there is a positive STR, there are low dropout rates. Some of the articles stated that STR did not affect dropping out directly. Lessard et al. (2010) stated that STR is related to a student’s academic achievement and satisfaction levels, which are in turn are associated with dropout rates. Also, a study by Wang et al. (2013) stated that STR was able to reduce dropout rates by mitigating the effects of negative peer pressure and conflicted parent–child relationships. It does not matter whether STR varies across school size and sector (Lee and Burkam, 2003), or if it has direct or indirect effects on dropout cases, what matters is that by using this research, education stakeholders can use STR as a measure of expected dropout rates. This association is significant, such that research done on the intention to leave came to the same conclusion (Frostad et al., 2014). Lee and Burkam (2003) also stated that positive relationships with staff and administrators was equally impactful as STR. Barile et al. (2011) went further and stated that evaluation of teachers by students led to a positive STR climate.

### Academic Curriculum and Student Achievement

Students who achieved good levels of academic scores were reported as less likely to drop out of schools (Lee and Burkam, 2003; Lessard et al., 2010; Cittone and Villani, 2019; Hou et al., 2021). To explain the cause and effect of academics on dropout rates, Lessard et al. (2010) associated an increase in academic achievement to an increase in commitment by the student. Dropout rates are not only affected by the academic success of the student but also by the teaching curriculum adapted by the school. Lee and Burkam (2003) found that schools offering mathematics courses had 28% lower dropout rates. The aim of the school curriculum should be to keep students ‘comfortably’ busy by not overstraining them but also not giving them too much free time.

Another curriculum factor was the administering of preschool education to students (Temple et al., 2000). Students who received pre-school education had a 24% less risk of dropping out compared to those in the control group. The causal relation is that preschool education reduces grade retention, frequent school mobility and increases parental involvement.

### School Structure and Organisation

Some of the school characteristics that were evaluated were race composition, gender composition, school size and sector. Christle et al. (2007) and Lee and Burkam (2003) both found that schools with a low-percentage of white students experienced reduced rates of dropouts. The reason for this is not well-known, but Welsh (2001) and Gottfredson et al. (2005), on the other hand, agreed that schools with higher ratios of African–American and Hispanic students had high levels of misconduct. The level of misconduct was later on linked by Archambault et al. (2009) and Loukas et al. (2010) to levels of dropout.

Schools with high, very high and low number of students experience higher risks of child dropout (Lee and Burkam, 2003; Christle et al., 2007; Marx et al., 2017; Fu et al., 2022). Even though Christle et al. (2007) stated a statistically insignificant association, other reviews (Prevatt and Kelly, 2003; Christenson and Thurlow, 2004) found a relationship between school size and risks of dropping out. The reason given was that high population negatively affects academic achievement, STRs and social deviance for the students.

In analysing school structures, the issue of school policies came up in Barile et al. (2011), with policies like teacher evaluation and rewarding teachers were evaluated. The researchers cautioned against awarding achieving students to ‘good’ teachers, since this causes a negative STR environment. Furthermore, Lee and Burkam (2003) reported that public schools faced more dropout rates than private schools.

### Student Emotional and Mental Health

Most governments in the world categorise education as a basic need; however, some school factors may hinder this requirement. The emotional and mental state of a student can be affected by factors within or outside school. Emotional wellbeing was evaluated in terms of school connectedness (LaRusso et al., 2007; Loukas et al., 2010; Wang et al., 2013; O’Reilly et al., 2018; Piñeiro-Cossio et al., 2021; Tsukawaki and Imura, 2022), family relations (Wang et al., 2013), loneliness (Frostad et al., 2014), and school engagement (Jimerson et al., 2003; Archambault et al., 2009). For example, in assessing students’ frame of mind, Archambault et al. (2009), used three aspects of school engagements: a measure of how much students liked school, and affective and behavioural engagement. School engagement predicted dropouts with a statistically significant correlation ratio of 0.15.

Most researchers concluded that mental health was connected to conduct problems, which were in turn connected to dropout rates. Hess and Copeland (2001) stated that students who sought more professional psychiatric help had a positive association with misbehaviour and were more likely to drop out of school. Emotional stability is an important aspect of a student’s wellbeing.

### Student Misconduct

Most studies associated dropping out with the increase in behaviour disorder (Hess and Copeland, 2001; Ryan and Patrick, 2001; Welsh, 2001; Gottfredson et al., 2005; Coetzee et al., 2022; Fernandez and Benner, 2022). There was significant positive association between dropout rates and law violation, suspension, and board violation rates (Christle et al., 2007). In the study by Hess and Copeland (2001), the researchers reported that students who showed high levels of stress had equally high levels of disorderly and risky behaviour involvement, and were more likely to drop out of school.

### Social Deviance

There were a few studies that evaluated the causes behind social deviance and misbehaviour in schools, presented as follows: Hess and Copeland (2001); Welsh (2001), Gottfredson et al. (2005), Murray and Malmgren (2005), Loukas et al. (2006, 2010), LaRusso et al. (2007), Gobat et al. (2021) and Chan et al. (2022). The reasons for behavioural problems in schools are school climate, student characteristics, and emotional and mental health of students.

### School Climate

There are a lot of factors that define the school climate, as illustrated by Welsh (2001), Gottfredson et al. (2005), Johns et al. (2019) and Hunter et al. (2022). Tables 3, 4 show the association between these factors and student behaviour deviance. Some of the most important factors that led to social misbehaviour are disrespect for student’s views and perspectives, unfairness and lack of clarity in school rules, poor school administration, poorly organised schools, high number of students, and lack of morale by teachers (Welsh, 2001; Gottfredson et al., 2005). The lack of morale, means that teachers are less involved with students and do not teach out of passion and rather treat it as a job.

**Table 3:** Show of associations between school climate, student characteristics and behaviour problems.

School climate and student characteristics	Behaviour problems				
	Offending	Misconduct	Victimisation	Avoidance	Feelings of safety
Respect for students	NA	NA	NA	NA	PA
School planning and action	NE	NE	NA	NE	PA
Fairness of rules	NA	NA	NA	NA	PA
Clarity of rules	NA	NA	NA	NA	PA
Student influence:	NA	NA	NE	PA	PA
Age	PA	NE	NA	NA	PA
Race (majority of students are non-white)	PA	PA	NA	NE	NE
Gender (majority of students are female)	NE	NA	NA	NA	PA
Involvement in school activities	PA	PA	PA	PA	NA
Positive peer associations	NA	NA	NA	NA	PA
Belief in school rules	NA	NA	NA	NA	PA

PA, positive association; NA, negative association; NE, no effect.

**Table 4:** Show of associations between school climate and school disorders.

School climate	School disorders		
	Teacher victimisation	Student victimisation	Student delinquency
School size	PA	PA	PA
Gender (majority of students are male)	PA	PA	PA
Race (majority of students are African–American)	PA	PA	PA
Fairness in school rules	NE	NA	NA
Clarity of rules	NE	NA	NA
Psychosocial climate	NA	NE	NE

PA, positive association; NA, negative association; NE, no effect.

### Students’ Emotional and Mental Health

Students’ misconduct are mainly results of emotional and mental issues. Issues like depressive traits (LaRusso et al., 2007), feeling of social isolation (LaRusso et al., 2007; Loukas et al., 2010), lack of interest in schools (Loukas et al., 2006, 2010) and high stress levels (Hess and Copeland, 2001).

### School-Based Student Behavioural Characteristics

Apart from emotional issues, other student factors that promote misbehaviour are increase in age, high student retention rate, high ratio of African–American or Hispanic students, high ratio of male students and students spending more time in school activities (Welsh, 2001; Gottfredson et al., 2005; O’Donnell et al., 2022; Salceda et al., 2022). When students spend more time in non-academic activities, they have a lot of free time to indulge in breaking rules (Lee and Burkam, 2003).

### Promoting Wellbeing in Schools

With regard to the interventions outlined in these studies, Murray and Malmgren (2005) examine the effects of an intervention aimed at increasing adolescent–teacher relationship, finding an improvement of the STR. Kelly et al. (2021) investigated the efficacy of a mentoring program based on psycho-spiritual education and aimed at enhancing school wellbeing and school climate. They found positive effects of psycho-spiritual education on school wellbeing and perceived school climate. Finally, Temple et al. (2000) investigated the effects of students’ participation in the Chicago Child–Parent Centre and Expansion Program on school dropout, finding a decrease in in problematic behaviours and dropout rates.

## Discussion

School dropout could be defined as a multifactorial phenomenon (Johansson and Uhnoo, 2019) determined by several risk and protective factors that can hinder or enhance students’ wellbeing and academic performance (Ramsdal and Wynn, 2022). The main aim of this review was to identify these risk and protective factors highlighted in literature to usually inform teachers, parents, the general public and interventions that can foster students’ school engagement reducing drop out and social deviant behaviours.

From our results emerged that STR and students’ emotions and mental health represents the main factors in predicting students’ drop out and social deviant behaviours.

With regard to students’ emotions and mental health the studies included in this review showed that the social wellbeing of students has an effect on the student’s feelings of belonging to school (LaRusso et al., 2007; Frostad et al., 2014). In this regard, the social setting in a school should be able to mitigate negative emotions like feelings of incompetency, lack of involvement and dislike of schooling life, and consequently increase the commitment and interest to learn. In addition to this, Frostad et al. (2014) stated that loneliness, in contrast to other factors like gender, teacher support and academic achievement, had the strongest associations with the intention to leave. From this review has also emerged that a positive school climate helps in mitigating the effect of negative family life and peer pressure. If students do not feel comfortable in school and at home, they are most likely to turn to friends who will mislead them into misbehaviour. Schools are thus responsible for the school crimes committed by their students; if schools provide the right climate by creating a positive STR, they will be able to reduce rates of students engaged in misbehaviour and misconduct.

Psychosocial factors negatively intervene in enhancing the relationships between dropouts and students’ difficulty in managing educational issues (Finn, 1989; Kratochwill and Stoiber, 2000). Specifically, several studies on dropout, have highlighted the importance of relationships in school dropout processes (Ramsdal et al., 2018). Students who had been separated from their parents over longer periods of time, had struggled to find friends in school, had not supportive teachers, and had struggled with mental health issues reported higher levels of dropout and social deviant behaviours. In particular, positive teacher–child relationships were found to reduce the association between early mental health problems and school dropout (Holen et al., 2018). Relationships in general seem to play an important role in school dropout. Teacher support and loneliness, indeed, predict students’ intention to leave school (Frostad et al., 2014).

Our review identified also other factors related to dropout and social deviance such as school organisation and structure, student individual characteristics and academic achievements. However, according to research that has explored students’ perceptions about school dropout with qualitative interviews, the main challenge of these students is represented by the management of stress related to social situations associated with failure and humiliation (Ramsdal and Wynn, 2022). According to literature, it seems that they lack the necessary resources to cope with these social situation, and this contributed to prolonged stress and failure to maintain their educational goals (16, 17 and 29).

Finally, with regard to interventions outlined in the reviewed studies several important points could be highlighted. Murray and Malmgren (2005) recommended that teachers should be more involved with their students, while Kelly et al. (2021) recommended the enrolment of students into programmes teaching psycho-spiritual principles of universal mind, consciousness and thought. They stated that the teachings improved the student’s mental health. Temple et al. (2000) recommended preschool education to teach students about the importance of education and how to handle any education-related issues. In addition to this, schools should improve STR by establishing medium-populated schools (Lee and Burkam, 2003) and treating students equally, despite their academic success (Barile et al., 2011). The teachers should also be respectful to students and willing to regard a student’s perspective instead of harshly discriminating their mental capabilities. Some schools should also revaluate their school curriculum to exert just the right amount of academic stress on students. Social relations are an important part of any person’s life, hence, schools should find a way of improving positive peer interactions. For instance, low achievers should stop being discriminated against, rather teachers should help them in their area of interest, be it music or arts. Schools should also improve the psycho-social climate in schools (Gottfredson et al., 2005). Professional help for emotional issues like stress and depression should be made readily available at school. Furthermore, this review demonstrates that mental health is important to a student’s wellbeing (LaRusso et al., 2007; Archambault et al., 2009; Loukas et al., 2010; Wang et al., 2013; Frostad et al., 2014); hence, the school should provide readily available mental healthcare.

## Limitations

The findings of this review should be interpreted in light of the limitations of our own work. Only assessed English-language literature has been assessed and may, therefore, significant findings reported in other languages have been overlooked. Although an exhaustive search was conducted, a relevant search term may have been omitted and consequently relevant studies may have not been retrieved. Finally, although we attempted to screen the retrieved studies thoroughly, it is possible that some salient studies were overlooked. Nonetheless, to the best of our knowledge, this review is the first to systematically review predictors of school dropout and deviant behaviours at school.

## Conclusion and Implications of the Study

Just like any other part of life, education has its own challenges. School wellbeing majorly impacts students’ dropout rates and social deviance problems, with the causes being related to the school climate, STR to a great extent, the school structure and social interactions among students. Schools should know that their environments have a huge impact on the student’s mental health, hence if preventive measures fail, treatment should be readily available.

To date, no single effect of interventions aimed at increasing school completion has been found to be explained by one single factor within the various factors associated with the risk of dropping out of school, confirming the multidimensional nature of these variables (Ramsdal and Wynn, 2022).

This review aimed at reports on a number of factors that can affect a student’s dropping-out rate and social deviance, in comparison most earlier studies that have only focussed on one factor, usually inform the creation of preventive and supportive interventions.

Future research perspectives could focus on the use of psycho-educational intervention protocols, not only in the school context but also in the wider community context.

## Data Availability Statement

The datasets presented in this study can be found in online repositories. The names of the repository/repositories and accession number(s) can be found in the article/supplementary material.

## Author Contributions

PL: introduction and conclusion. All authors contributed to the article and approved the submitted version.

## Conflict of Interest

The authors declare that the research was conducted in the absence of any commercial or financial relationships that could be construed as a potential conflict of interest.

## Publisher’s Note

All claims expressed in this article are solely those of the authors and do not necessarily represent those of their affiliated organizations, or those of the publisher, the editors and the reviewers. Any product that may be evaluated in this article, or claim that may be made by its manufacturer, is not guaranteed or endorsed by the publisher.
